# Verifying the Usefulness of Anticancer Drug-Degrading Agents Using the Residual Amount of Fluorouracil After Wiping as an Indicator

**DOI:** 10.7759/cureus.77104

**Published:** 2025-01-07

**Authors:** Kanako Shimizu, Takahiro Amemiya, Syunsuke Kumazawa, Tomoharu Takeda, Takashi Tomita, Masanari Takahashi

**Affiliations:** 1 Department of Pharmacy, International University of Health and Welfare, Mita Hospital, Minato-ku, JPN; 2 Department of Pharmacy, Sanno Hospital, Minato-ku, JPN; 3 Department of Pharmaceutical Sciences, School of Pharmacy at Narita, International University of Health and Welfare, Narita-shi, JPN; 4 Products and Process Analysis Technology Laboratory, Chemical Analysis Technology Center, Toshiba Nanoanalysis Corporation, Yokohama-shi, JPN; 5 Department of Pharmacy, Hitachiomiya Saiseikai Hospital, Hitachiomiya-shi, JPN

**Keywords:** anticancer drug, anticancer drug degrading agents, fluorouracil, hd protect, tripleclin

## Abstract

Long-term exposure to anticancer agents poses a health risk to healthcare workers and requires safety measures such as biological safety cabinets and personal protective equipment. The purpose of this study was to verify the usefulness of two anticancer drug-degrading agents, HD Protect (Secom Medical System Co. Ltd., Tokyo, Japan) and Tripleclin (Nipro Co. Ltd., Settsu, Japan), by analyzing residual amounts of fluorouracil (5-FU), which is frequently used in real clinical practice. Wiping with HD Protect and Tripleclin showed lower 5-FU residual concentrations than wiping with control in this study, indicating that they may be suitable for higher 5-FU concentrations. This study confirms the usefulness of wiping with anticancer drug-degrading agents to reduce 5-FU residues.

## Introduction

Long-term exposure to anticancer drugs is a potential health hazard for healthcare workers handling anticancer drugs [1]. Therefore, the use of biological safety cabinets (BSCs), closed-system drug transfer devices (CSTDs), and personal protective equipment (PPE) is recommended to reduce exposure to anticancer agents [2]. While these measures reduce exposure to anticancer agents in and around the preparation environment [3], they do not completely eliminate it. Contamination of gloves and adhesion of anticancer agents to infusion bags after preparation remain concerns [4]. Therefore, comprehensive cleaning measures need to be urgently developed to prevent exposure to anticancer agents in the medical environment.

Recently, to combat exposure to anticancer drugs, anticancer drug-degrading agents have been developed, including HD Protect (Secom Medical System Co. Ltd., Tokyo, Japan) and Tripleclin (Nipro Co. Ltd., Settsu, Japan), which are commercially available in Japan. HD Protect, a spray containing adjusted sodium hypochlorite and pH, is characterized by its non-irritant effects on the skin and eyes, minimal metal corrosiveness, and ease of use in stainless steel equipment [5]. Tripleclin is a three-wipe system impregnated with three agents: sodium hypochlorite, sodium thiosulfate, and sodium hydroxide. The three wipes are used in sequence for maximum effectiveness [6]. The type and amount of residual anticancer drugs are likely to differ among medical facilities owing to differences in regimens and anticancer drug doses. Furthermore, differences in the working processes of the anticancer drug degrading agent operators may lead to differences in anticancer agent degradation rates. However, these differences at different facilities have not been evaluated.

Therefore, to individualize anticancer drug-degrading agents for each facility as part of anticancer drug exposure countermeasures, we aimed to verify the usefulness of anticancer drug-degrading agents. We evaluated the residual amount of fluorouracil (5-FU), which is indicated for various cancer types and frequently used in clinical practice, after wiping with anticancer drug-degrading agents. This study provides important insights into the usefulness of anticancer drug-degrading agents for other anticancer drugs.

## Technical report

Methods

Materials

The 5-FU solution (250 mg/5 mL) was procured from Kyowa Kirin Co., Ltd., Tokyo, Japan (lot. 22801SF and 23501SF).

Preparing 5-FU Coated Stainless Steel Plates

Fluorouracil was prepared at concentrations of 1000 ppm (µg/mL) and 10 ppm (µg/mL) by diluting in saline solution (lot. M2B76 and K4C89; Otsuka Pharmaceutical Co., Ltd., Tokyo, Japan). Ten μL of each solution was evenly applied to a 10 cm × 10 cm stainless steel plate and repeated 10 times. The dropped stainless steel plates were left to dry for three hours at room temperature to produce adhesive surfaces containing 100 and 1 μg of 5-FU.

Procedure for Wiping Operation With Anticancer Drug-Degrading Agents

The 5-FU-coated stainless steel plate was sprayed with sufficient HD Protect four times and immediately wiped with a Kim towel (Nippon Paper Crecia Co., Ltd, Tokyo, Japan). Alternatively, the stainless steel plate was initially wiped with a sodium hypochlorite sheet in Tripleclin for one minute. The area was wiped with a sodium thiosulfate sheet and left to stand for one minute. The area was then wiped with a sodium hydroxide sheet and allowed to stand for one minute. Finally, the area was wiped using a Kim towel moistened with distilled water (lot. K3A74 and K4C88; Otsuka Pharmaceutical Co., Ltd.). As a control, the stainless steel plate was wiped off with a Kim towel moistened with Otsuka distilled water, then sprayed with Cucute CLEAR Foam Spray® as a neutral detergent (Kao Co., Ltd., Tokyo, Japan) and wiped with a Kim towel moistened with Otsuka distilled water. This process was repeated once more. Finally, wiping was performed using a Kim towel moistened with Otsuka distilled water.

Quantitative Analysis of 5-FU in Stainless Steel Plates

Each stainless steel plate was subjected to the wiping operation and the stainless steel plates for 5-FU addition and recovery were wiped with FINESPEC (Mitsubishi Chemical Co., Ltd., Tokyo, Japan) moistened with distilled water from Autostill WG711 (Yamato Scientific, Tokyo, Japan). The stainless steel plates were wiped by the same individual. The number of wiping operations and time were standardized by performing two wiping operations per stainless steel plate each time vertically and horizontally in 70 seconds. Extraction of the wiped material was performed immediately after wiping, and the extracted solution was stored at 4°C and measured within one day after extraction. Distilled water was subsequently added to FINESPEC and sonicated for five minutes using BRANSON 5800 (Yamato Scientific). The mixture was then stirred at high speed for five minutes using MSV-3500 (Biosan Ltd., Riga, Latvia) and filtered through Millex-LG (Merck KGaA, Darmstadt, Germany) to obtain the sample solution. The 5-FU concentration in the sample solution was analyzed using liquid chromatography-mass spectrometry (LC-MS/MS; ACQUITY UPLC, Xevo TQ-XS, Waters, Milford, MA) with 5-bromouracil (Tokyo Chemical Industry Co., Ltd., Tokyo, Japan) as an internal standard. The detection limit for 5-FU determination by LC-MS/MS is 0.1 ng/mL. The analytical conditions were as follows: column: ACQUITY UPLC BEH Shield RP18 (2.1 × 100 mm); mobile phase: A:B = acetonitrile/water (4:1, v/v); flow velocity: 0.4 mL/min; ionization: electrospray ionization (ESI) (-); and monitor ion (m/z): 127.8→41.9.

Statistical Analysis

IBM SPSS Statistics version 26 (IBM, Armonk, NY) was used for statistical examination. Tukey-Kramer test was used to compare residuals after using HD Protect, Tripleclin, or control method. P < 0.05 was considered statistically significant.

Results

Addition-Recovery Rate Using 5-FU

Initially, an addition-recovery experiment was conducted using 1 μg and 100 μg of 5-FU to validate the assay system (Table 1). The recovery of 5-FU was over 99% for both concentrations, suggesting the validity of the assay system.

**Table 1:** Addition and recovery experiments with fluorouracil (5-FU). One μg and 100 μg of 5-FU were added to stainless-steel plates and the residual amount of 5-FU recovered using FINESPEC was measured and the recovery rate was calculated. Recovery rate (%) = 5-FU residual amount/5-FU added x 100. * Only values greater than 100 are presented in three-digit numbers.

5-FU added	Number	5-FU residual (μg)	5-FU removal recovery rate (%)
1 μg	1	0.99	99
	2	1.1	109
100 μg	1	104^*^	104^*^
	2	110^*^	110^*^

Verification of the 5-FU Residual Amount After Wiping With Anticancer Degrading Agents

After using HD Protect and Tripleclin as anticancer drug-degrading agents and a control technique, 5-FU residual amounts were assessed (Table 2). Residual levels at 1 μg 5-FU were virtually unobserved after the use of HD Protect and Tripleclin, and in the control (wipe sheets moistened with injectable water). Furthermore, at 5-FU 100 μg, the control plate had higher trace residuals than plates wiped with HD Protect or Tripleclin. In addition, values could not be directly compared using statistical methods because < 0.001 µg of 5-FU was included as a residual.

**Table 2:** Evaluating fluorouracil (5-FU) wiping removal using anticancer degradation agents. 5-FU added to the stainless-steel plate was subjected to a wipe-off operation using HD Protect, Tripleclin, and a control method. The amount of 5-FU remaining on the stainless steel plate after each wiping operation was measured.

Anticancer drug-degrading agent	5-FU added	Number	5-FU residual (μg)
HD Protect	1 μg	1	<0.001
		2	<0.001
		3	<0.001
	100 μg	1	0.003
		2	0.003
		3	0.003
Tripleclin	1 μg	1	<0.001
		2	<0.001
		3	<0.001
	100 μg	1	0.005
		2	<0.001
		3	<0.001
Control	1 μg	1	<0.001
		2	<0.001
		3	<0.001
	100 μg	1	0.025
		2	0.023
		3	0.034

## Discussion

In this study, anticancer drug removal is for the first time evaluated using the same experimental system and two anticancer drug-degrading agents. While HD Protect and Tripleclin websites contain data on the degradation rates of various anticancer agents, they lack comprehensive descriptions of the experimental methods [5,6]. Standardization of wiping operation and time is useful to ensure the consistent effectiveness of anticancer drug-degrading agents in clinical practice. Therefore, we verified the wiping operation and time for HD Protect and Tripleclin, commercially available anticancer drug-degrading agents. Low 5-FU concentrations were almost completely degraded using HD Protect, Tripleclin, and control methods. However, at high 5-FU concentrations, the control group tended to leave more 5-FU residues than the HD Protect or Tripleclin groups, suggesting that wiping with these agents may be more suitable. The amount of anticancer drug adherence may vary by facility location and the type and dosage of anticancer drugs used, making it difficult to accurately determine adherence concentrations. Nevertheless, as 5-FU has been detected at levels ranging from undetectable to approximately 2 μg across various facilities, we set the low concentration as 1 μg and the high concentration as 100 μg, two orders of magnitude higher [7]. Moreover, HD Protect and Tripleclin used in this study contain sodium hypochlorite (NaOCl) [5,6], which is a strong oxidant that degrades anticancer agents, such as epirubicin, cisplatin, carboplatin, and cyclophosphamide [8], but is corrosive to humans and metals. However, the concentration and pH of NaOCl in HD Protect used in this study were adjusted and considered safe [5].

Identifying residual anticancer agents in the medical environment and accurately assessing their amount is crucial for selecting the most appropriate anticancer degrading agent for each facility. The recovery rate of anticancer drugs from the healthcare environment is a useful indicator for assessing anticancer drug residue levels. In this study, the recovery rate of 5-FU was over 99%, suggesting that the recovery technique used was appropriate. A previous study using lactose hydrate as an alternative sample for hazardous drugs achieved a recovery rate exceeding 80% using lactose hydrate added to stainless plates [9], indicating the usefulness of the technique. As we applied the same recovery method in this study, the number of measurements was deemed appropriate. The measurements were generally consistent in this study, although a variation was observed for 100 μg of 5-FU with Tripleclin. This result was attributed to slight differences in the wiping techniques of the workers, as the manipulation process with Tripleclin constituted three wiping phases. Therefore, HD Protect, requiring only one wiping operation after spraying, exhibited less variability in the measured values. Moreover, as the spray-type HD Protect, which is easy to operate in clinical settings, may be more versatile, including it in spill kits used for exposure controls in the event of anticancer drug leakage in hospitals could be beneficial [10].

A limitation of this study is that we only analyzed 5-FU, which is frequently used in clinical practice. The residual amounts needed for other anticancer agents should be verified at each medical facility when applied.

This study has two novel aspects. First, we standardized wiping with anticancer drug-degrading agents; wiping for residual 5-FU recovery and time for 5-FU, which is frequently used in clinical practice and indicated for various cancer types. This standardization is important for verifying the usefulness of anticancer drug-degrading agents for other anticancer drugs in the future. Second, the wiping operation was verified using a stainless steel plate, which is an experimental method consistent with actual clinical practice, assuming that the anticancer drug was spilled inside a safety cabinet. We will consider the importance of the number of times and duration of the wiping operation in examining the use of anticancer drug-degrading agents in other environments in future research.

## Conclusions

Residual levels of 5-FU at 1 μg were virtually unobserved after the use of HD Protect, Tripleclin, and the control. Furthermore, for 5-FU at 100 μg, the control plate had higher trace residuals than plates wiped with HD Protect or Tripleclin. This study demonstrated that wiping with HD Protect or Tripleclin may be more suitable for high 5-FU concentrations with lower 5-FU residual levels than the control method. Hence, we demonstrated the usefulness of the wiping operation with anticancer degrading agents for 5-FU.
